# Efficacy of optical coherence tomography in the triage of women with minor abnormal cervical cytology before colposcopy

**DOI:** 10.1371/journal.pone.0282833

**Published:** 2023-03-13

**Authors:** Lei Yan, Xiao Xiao, Ling He, Liye Shi, Xue Yang, Jun Zhang, Yongjing Zhang, Chun Fu

**Affiliations:** Department of Obstetrics and Gynecology, The Second Xiangya Hospital of Central South University, Changsha, Hunan, China; Women’s Hospital, School of Medicine, Zhejiang University, Hangzhou, China, CHINA

## Abstract

**Objectives:**

To evaluate the efficacy of Optical Coherence Tomography (OCT) for detecting cervical lesions in women with minor abnormal cytology results (atypical squamous cells of undetermined significance (ASC-US) and low-grade squamous intraepithelial lesion (LSIL)).

**Methods:**

A prospective study was conducted at gynecologic clinic from Mar 2021 to Sep 2021. The recruited women with cervical cytological findings of ASC-US or LSIL were inspected with OCT before colposcopy-directed cervical biopsy. The diagnostic performance of OCT, alone and in combination with high-risk human papillomavirus (hrHPV) testing were evaluated to detect cervical intraepithelial neoplasia of grade 2 or worse (CIN2+)/CIN3 or worse (CIN3+). The rate of colposcopy referral and the immediate risk of CIN3+ of OCT were calculated.

**Results:**

A total of 349 women with minor abnormal cervical cytology results were enrolled. For detection of CIN2+/CIN3+, the sensitivity and NPV of OCT were lower than those of hrHPV testing (CIN2+: 71.3% vs. 95.4%, 89.0% vs. 91.1%, *P* < 0.001; CIN3+: 75% vs. 93.8%, 96.5% vs. 95.6%, *P* < 0.001), but the specificity, accuracy and PPV were higher than those of hrHPV testing (CIN2+: 77.5% vs. 15.6%, 75.9% vs. 35.5%, 51.2% vs. 27.3%, *P* < 0.001; CIN3+: 69.4% vs. 13.6%, 69.9% vs. 20.9%, 19.8% vs. 9.9%, *P* < 0.001). OCT combined with hrHPV testing (CIN2+: 80.9%; CIN3+: 72.6%) showed higher specificity than that of OCT alone (*P* < 0.001). The colposcopy referral rate base on OCT classification was lower than that based on hrHPV testing (34.7% vs. 87.1%, *P* < 0.001). Patients with hrHPV-positive ASC-US and hrHPV-negative LSIL cytology, the immediate CIN3+ risk in OCT negative cases was less than 4%.

**Conclusions:**

OCT alone or combination with hrHPV testing shows good performance for detecting CIN2+/CIN3+ in patients with ASC-US/LSIL cytology. OCT is an effective method for colposcopy triage in women with hrHPV-positive ASC-US and hrHPV-negative LSIL cytology.

## Introduction

Cervical cancer is one of the most common malignant tumors in female reproductive system. Effective screening of cervical precancerous lesions is currently the most important method to prevent cervical cancer in China [1, 2]. The cervical cytology and high-risk human papillomavirus (hrHPV) testing alone or co-testing are now recommended as primary cervical screening strategies [3, 4]. The diagnostic value of ThinPrep^®^ Cytologic Test (TCT) combined with hrHPV testing can increase the sensitivity and negative predictive value (NPV) of cervical screening [5, 6]. However, management recommendations for minimally abnormal cervical cancer screening test results are still controversial [7]. Minor abnormal cervical cytology results include atypical squamous cells of undetermined significance (ASC-US) and low-grade squamous intraepithelial lesion (LSIL) [7, 8]. Colposcopy is recommended for individuals with hrHPV-positive ASC-US test result or LSIL test result as the guidelines [3]. In fact, the proportion of histopathological diagnosis of cervical intraepithelial neoplasia grade 2/3 or worse (CIN2/3+) is low in ASC-US or LSIL cases [9, 10]. Therefore, combining the novel methods with conventional cervical screening strategies may be of great value for accurately screening cervical cancer and avoiding unnecessary referrals for colposcopy.

Optical coherence tomography (OCT) is a biomedical optical imaging method with the advantages of high-resolution, non-invasiveness and real-time imaging. The optical ultrasonic detector uses the principle of near-infrared light interference to obtain the OCT images containing epithelial and stromal structure. It can detect cervical tissue with a resolution of 3–20 μm and a penetration depth of 2 mm [11, 12]. After 20 years of development, the diagnostic efficiency of OCT for cervical lesions has been confirmed by in vitro and in vivo tests, and the image reading standard has been formulated [13, 14]. Studies indicates that OCT is an auxiliary means of colposcopy in the diagnosis of cervical lesions [11, 15, 16]. Therefore, OCT combined with cervical screening methods may provide new ideas for precise screening of cervical cancer.

In this study, we obtained OCT images of in-vivo cervical tissues of women with minor abnormal TCT results of ASC-US and LSIL. Then, we evaluated the efficiency of OCT alone and combination with hrHPV testing to detect CIN2+/CIN3+. Finally, we explored the value of OCT in the triage of women with minor abnormal cervical cytology before colposcopy.

## Materials and methods

### Participants and study design

The study was conducted in accordance with the Declaration of Helsinki Ethical Principles and was approved by the Ethics Committee of the Second Xiangya Hospital of Central South University (LYF-2021026). 406 gynecological outpatients with abnormal cytological results were recruited at the Second Xiangya Hospital of Central South University from Mar 2021 to Sep 2021.

**Fig 1:**
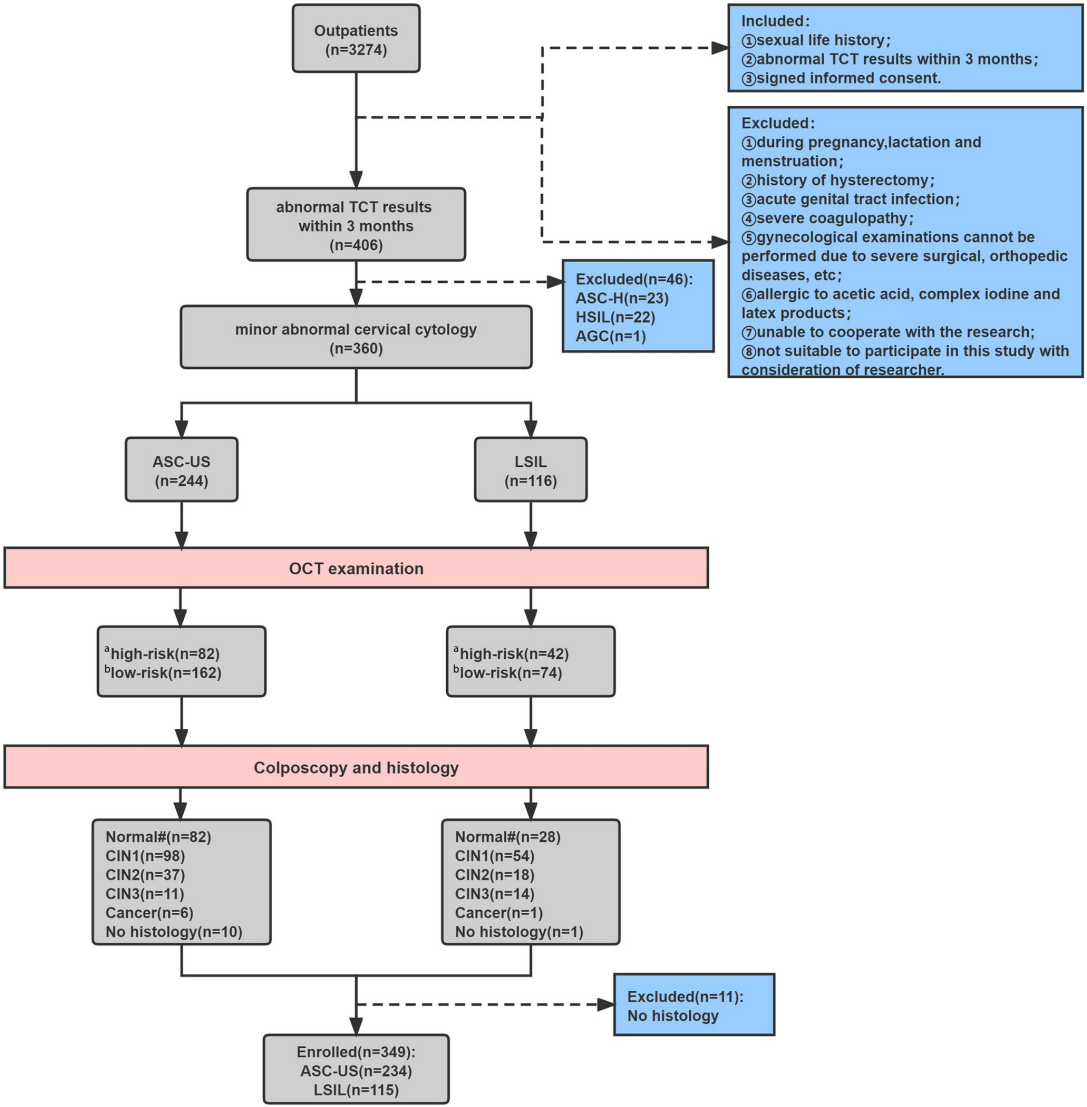
The study flowchart and study outcomes. ^a^, high-risk: we defined high-grade squamous intraepithelial lesion (HSIL) and invasive lesion as high-risk or positive. ^b^, low-risk: we defined normal/mild inflammation, ectropion, and low-grade squamous intraepithelial lesion (LSIL) as low-risk or negative. ^#^, No intraepithelial lesion was found in biopsy. ASCUS, atypical squamous cells of undetermined significance; LSIL, low-grade squamous intraepithelial lesion; ASC-H, atypical squamous cells cannot exclude high-grade squamous intraepithelial lesion; HSIL, high-grade squamous intraepithelial lesion; AGC, atypical glandular epithelial cells; OCT, optical coherence tomography; CIN, cervical intraepithelial neoplasia.

Women with minor abnormal TCT results of ASC-US or LSIL were included in the study. All participants were required to have the results of TCT and hrHPV tests within 3 months. The exclusion criteria were as follows: during pregnancy or lactation or menstruation; history of hysterectomy; acute genital tract infection; severe coagulation disease; unable to perform gynecological examination; allergic to acetic acid, complex iodine and latex products; unable to cooperate with the research; not suitable to participate in this study with consideration of researcher; no histological results; TCT results of atypical squamous cells cannot exclude high-grade squamous intraepithelial lesion (ASC-H), high-grade squamous intraepithelial lesion (HSIL) and atypical glandular epithelial cells. A written informed consent was obtained from every participant. After signing informed consent, all participants were inspected with in-vivo OCT examination before colposcopy-directed cervical biopsy. The study flowchart was illustrated in Fig 1.

### Cervical cytology examination

ThinPrep^®^ Cytologic Test (TCT, Hologic, Marlborough, MA, USA) was used to determine the cervical cytology testing. The Bethesda System (TBS) for reporting cervical cytology in 2014 was applied for TCT report format [17].

### HrHPV testing

HrHPV testing (HybriBio, Guangdong, China) was performed according to the instructions of manufacturer. The principle of test is flow-through hybridization and gene chip. The test kit detects 21 HPV genotypes, including 15 high-risk types (types 16, 18, 31, 33, 35, 39, 45, 51, 52, 53, 56, 58, 59, 66 and 68) and 6 low-risk types (types 6, 11, 42, 43, 44, and CP8304 [18]).

### Clinical OCT system

Ultralucia OCT Cervical Scanning System (model: UL-C110) was developed by Zhengzhou Ultralucia Medical Technology Co., Ltd. It consists of laser light source, spectrum imager, interferometer, imaging processing system, computer, display, reference arm and handheld probe. Based on the principle of low-coherence light interference, the OCT system detects the interference signals formed by reference light and backscattered light from different depths of the sample to achieve the images of shallow biological tissue. The near-infrared wavelength of device is in the range of 750–950 nm. The wavelength can provide an axial resolution of < 5 μm and a transverse resolution of < 10 μm in tissue. A handheld probe is used to deliver the imaging beam to the surface of cervix. The optical power of the probe output beam is < 5 mW, and the imaging depth is 2 mm. The device system can complete in vivo scanning of 12 locations of cervical tissue in 2–3 minutes and obtain micron images.

### OCT examination

**Fig 2:**
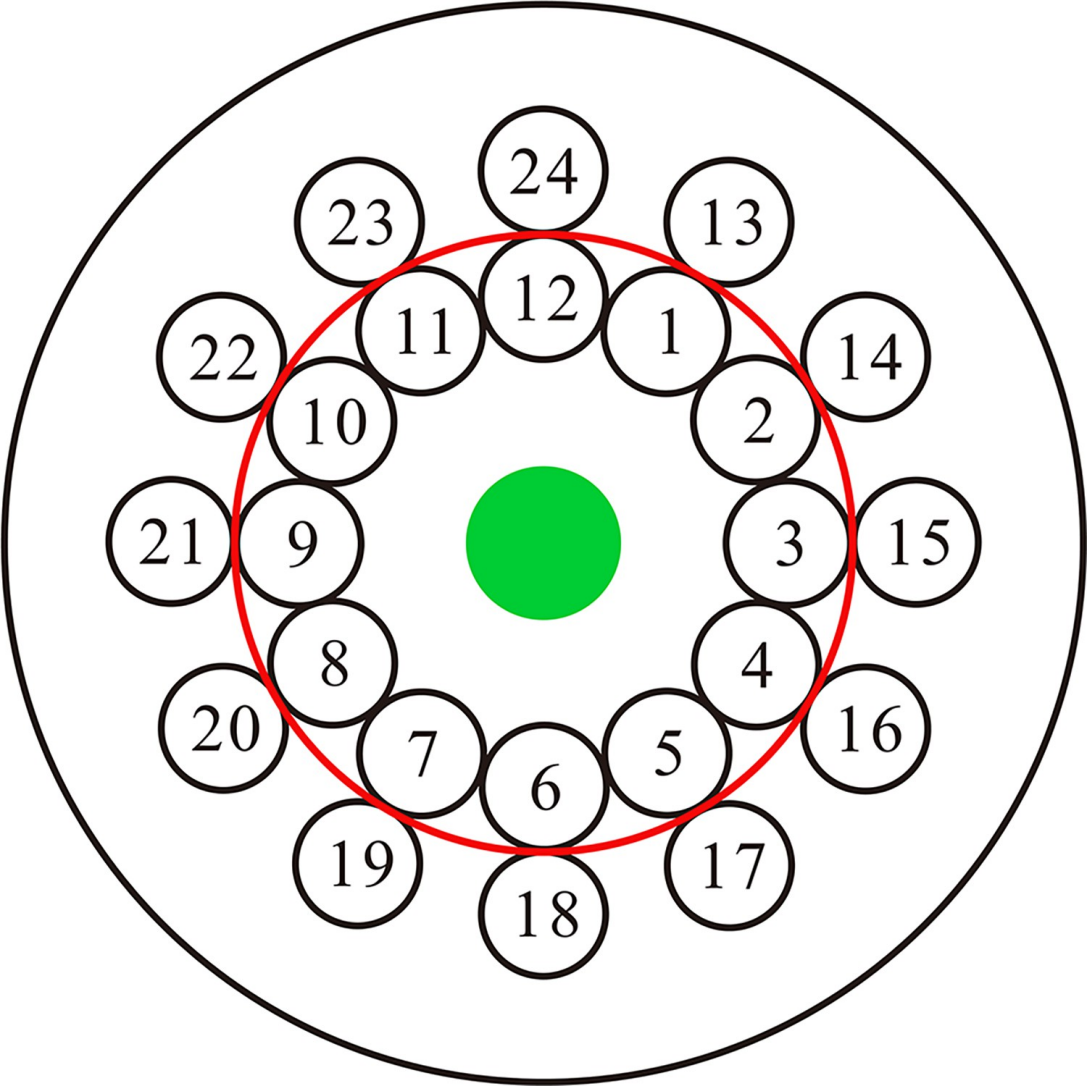
OCT scanning schematic diagram. The gynecologist scanned 24 locations (12 locations in the inner circle and 12 locations in the outer circle in clockwise direction) on cervical surface and saved the OCT images. Green circular area: cervical canal. Red circular strip: squamocolumnar junction (SCJ).

OCT examinations were performed by one experienced gynecologist with OCT training. The participant lay down in the lithotomy position, then the gynecologist inserted a speculum into the participant’s vagina to fully expose the cervix. After wiping off secretion and making a preliminary visual observation, the gynecologist used the handheld probe of the OCT system to inspect the cervical surface. The gynecologist scanned 24 locations (12 locations in the inner circle and 12 locations in the outer circle in clockwise direction) on cervical surface and saved the OCT images (Fig 2). The diagnosis of OCT images was performed before colposcopy-directed cervical biopsy. The probe was mounted a disposable latex protective cover, which had no visual influence on the imaging. The operator kept selected locations of the cervical tissue in the 200 μm working distance of the probe.

### OCT image processing and comparison

The feature interpretation of OCT images was based on the classification criteria described by Ren [14]. The images were classified into two categories: normal/mild inflammation, ectropion, and LSIL as OCT images of low-risk or negative; HSIL and invasive lesion as OCT images of high-risk or positive. To improve the diagnostic accuracy of OCT images, two trained investigators (a gynecologist and a technician) used image processing software (ImageJ, version 1.52a) to review the OCT images. Only patient information of the age, menstruation, and hrHPV/TCT results were provided to the investigators. For each participant, a positive result was given if at least one image at one location was marked as high-risk. Otherwise, a negative result was given. If two investigators had different opinions, an agreement will be reached through discussion.

### Colposcopy and histology

All participants received colposcopy (3ML LED, Leisegang, Berlin, German) and cervical biopsy. The colposcopist determined the biopsy sites. Firstly, the colposcopist observed the whole picture of the vagina and cervix. The cervical observation sites were focused on cervical squamocolumnar junction (SCJ) and transformation zone (TZ). Secondly, the colposcopist took biopsies at the suspicious locations based on the OCT diagnosis and colposcopy impression, or took multiple biopsies to improve the diagnostic accuracy of atypical cases. Endocervical curettage (ECC) was performed in cervix with TZ II or III. Finally, the biopsy samples were examined pathologically on the Second Xiangya Hospital of Central South University. Histological results were reported using the CIN classification system [18]. The pathological results of cervical tissue were the gold standard for diagnosis.

### Statistical analysis

Basic information of the participant included age, menstruation, treatment history, personal history, TCT, hrHPV, OCT, colposcopy, and pathological results. All data were recorded in a dedicated Excel file. The diagnostic performance of OCT and hrHPV testing for CIN2+ and CIN3+ detection were evaluated by calculating the sensitivity, specificity, accuracy, positive predictive value (PPV), NPV, positive likelihood ratio (+LR), negative likelihood ratio (-LR) and their 95% confidence interval (CI). The mean and standard deviation were calculated for the categorical variables. Categorical variables were compared using chi-square test and Cochran’s Q test. All statistical analyses were performed using the SPSS software (version 26.0, IBM, Armonk, NY, USA), and a p-value of less than 0.05 was considered statistically significant.

## Results

### Participant characteristics and clinical outcomes

A total of 349 participants with histopathological results were enrolled, 234 cases were ASC-US and 115 cases were LSIL (Fig 1 and Table 1). The mean age was 45 (range, 20–76) years. There were 110 normal cervixes or chronic cervicitis, 152 CIN1, 55 CIN2, 25 CIN3 and 7 cervical cancers. 76.9% (180/234) and 71.3% (82/115) of participants had a pathological diagnosis of ≤ CIN1.

**Table 1:** Participants’ characteristics and clinical outcomes.

Index	ASC-US	LSIL	Total
	n (%)	n (%)	n (%)
**Total**	234	115	349
**Mean age (range)**	45.10±11.06(20–76)	44.30±11.31(21–68)	44.83±11.13(20–76)
**Menstruation**			349
Non-menopause	156(66.7)	80(69.6)	236(67.6)
Post-menopause	78(33.3)	35(30.4)	113(32.4)
**OCT**			349
high-risk^a^	79(33.8)	42(36.5)	121(34.7)
low-risk^b^	155(66.2)	73(63.4)	228(65.3)
**hrHPV**			349
HPV 16/18 (+)	46(19.7)	22(19.1)	68(19.5)
HPV 16/18 (-)^§^	154(65.8)	82(72.2)	236(67.6)
hrHPV (-)	34(17.5)	11(9.6)	45(12.9)
**Pathology**			349
Normal^#^	82(35.0)	28(24.3)	110(31.5)
CIN1	98(41.9)	54(47.0)	152(43.6)
CIN2	37(15.8)	18(15.7)	55(15.8)
CIN3	11(4.7)	14(12.2)	25(7.2)
Ca	6(2.6)	1(0.9)	7(2.0)

TCT, ThinPrep® Cytologic Test; ASCUS, atypical squamous cells of undetermined significance; LSIL, low-grade squamous intraepithelial lesion; OCT, optical coherence tomography; hrHPV, high-risk human papillomavirus; HPV16/18, HPV-16 or HPV-18; CIN, cervical intraepithelial neoplasia; Ca, cancer.

^a^, high-risk: we defined HSIL and invasive lesion as high-risk or positive.

^b^, low-risk: we defined normal/mild inflammation, ectropion, and LSIL as low-risk or negative.

^§^, HPV16/18 (-): hrHPV positive result excluded HPV-16 and HPV-18 positive result.

^#^, No intraepithelial lesion was found in biopsy.

For each participant, we recorded all hrHPV DNA test results from within 5 years prior to enrollment. We found that 207 participants had received HPV DNA testing, 33 cases were HPV16/18 positive, 125 cases were non HPV16/18 positive and 49 cases were hrHPV-negative.

The number of participants with OCT images of high-risk was 121 (34.7%). Their pathological diagnosis was 19 normal, 40 CIN1, 38 CIN2, 19 CIN3 and 5 cancers, respectively. The number of hrHPV-positive women was 304 (87.1%). The pathological diagnosis was 90 normal, 131 CIN1, 53 CIN2, 24 CIN3 and 6 cancers, respectively.

### Diagnostic features of OCT images

All participants obtained cervical OCT images and colposcopy images. Cervical OCT images can show the three-layered structure of cervical epithelium, basement membrane and stroma. We evaluated OCT images based on refractive uniformity, epithelial integrity, basement membrane visibility and stroma structure.

Low-risk cervical OCT images include normal cervical tissue (S1A1 Fig), Nabothian cysts (NC) (S1B1 Fig), cervical columnar epithelial ectropion (S1C1 Fig), and LSIL (S1D1 Fig) in the study. High-risk cervical OCT images include HSIL (S2A1 Fig) and cervical cancer (S2B1 Fig). We found some special feature of OCT images, such as patients after the treatment of loop electrosurgical excision procedure (LEEP) (S3A1 Fig) and atrophic vaginitis before and after estrogen treatment (S3B1 and S3C1 Fig).

### Comparison of the performance of OCT and hrHPV testing for detecting CIN2+/CIN3+

Of the 87 CIN2+ participants, 62 (71.3%) were detected by OCT and 83 (95.4%) by hrHPV testing. OCT co-testing with hrHPV testing detected 59 (67.8%) CIN2+ cases, and OCT or hrHPV testing detected 86 (98.9%) CIN2+ cases (Table 2). Among 32 CIN3+ patients, 24 (75%) were detected by OCT and 30 (93.8%) by hrHPV testing. OCT co-testing with hrHPV testing detected 22 (68.8%) CIN3+ cases, and OCT or hrHPV testing detected 32 (100%) CIN3+ cases (Table 2).

**Table 2:** Positive rate of different screening methods.

Screening method (n)	Pathology				
	Normal^#^	CIN1	CIN2	CIN3	Ca
	n (%)	n (%)	n (%)	n (%)	n (%)
Subjects (349)	110(100)	152(100)	55(100)	25(100)	7(100)
OCT (+) (121)	19(17.3)	40(26.3)	38(69.1)	19(76.0)	5(71.4)
hrHPV (+) (304)	90(81.8)	131(86.2)	53(96.4)	24(96.0)	6(85.7)
OCT and hrHPV^†^ (+) (109)	15(13.6)	35(23.0)	37(67.3)	18(72.0)	4(57.1)
OCT or hrHPV^‡^ (+) (316)	94(85.5)	136(89.5)	54(98.2)	25(100)	7(100)
HPV16/18 (+) (68)	19(17.3)	19(12.5)	16(29.1)	10(40.0)	4(57.1)
OCT and HPV16/18^†^ (+) (29)	2(1.8)	3(2.0)	12(21.8)	9(36.0)	3(42.9)
OCT or HPV16/18^‡^ (+) (160)	36(32.7)	56(36.8)	42(76.4)	20(80.0)	6(85.7)

CIN, cervical intraepithelial neoplasia; Ca, cancer; OCT, Optical coherence tomography; hrHPV, high-risk human papillomavirus; HPV16/18, HPV-16 or HPV-18.

^#^, No intraepithelial lesion was found in biopsy.

^†^, “and”: the AND rule, in which the diagnosis is positive only if both A and B are positive. Either A or B can be negative for the diagnosis to be negative, trust negative.

^‡^, “or”: the OR rule, in which the diagnosis is positive if either A or B is positive. Both A and B must be negative for the diagnosis to be negative, trust positive.

To evaluate the diagnostic efficiency of OCT, we compared the sensitivity, specificity, accuracy, PPV, NPV, +LR and -LR of OCT, along and in combination with hrHPV testing for detecting CIN2+ and CIN3+ (Table 3). When cervical cytology was minor abnormal, the sensitivity and NPV of OCT were lower than those of hrHPV testing (CIN2+: 71.3% vs. 95.4%, 89.0% vs. 91.1%, respectively, *P* < 0.001; CIN3+: 75% vs. 93.8%, 96.5% vs. 95.6%, respectively, *P* < 0.001), but the specificity, accuracy, and PPV were higher than those of hrHPV testing (CIN2+: 77.5% vs. 15.6%, 75.9% vs. 35.5%, 51.2% vs. 27.3%, respectively, *P* < 0.001; CIN3+: 69.4% vs. 13.6%, 69.9% vs. 20.9%, 19.8% vs. 9.9%, respectively, *P* < 0.001). The +LR of OCT was higher than that of hrHPV testing (CIN2+: 3.165 vs. 1.131, *P* < 0.001; CIN3+: 2.451 vs. 1.085, *P* < 0.001). When hrHPV testing and OCT were at least one positive, they had highest sensitivity 98.9% among different screening methods for detecting CIN2+ (*P* < 0.001) and 100% for detecting CIN3+ (*P* < 0.001). When both hrHPV testing and OCT were positive, they had the highest specificity 80.9% (*P* < 0.001) for detecting CIN2+ and 72.6% (*P* < 0.001) for detecting CIN3+. The diagnostic efficiency of different screening methods for detecting CIN2+ and CIN3+ were shown in Table 3.

**Table 3:** The performance of different screening methods for detecting CIN2+/CIN3+.

TCT result	screening method		sensitivity(%)	specificity(%)	Accuracy	PPV(%)	NPV(%)	+LR(%)	-LR(%)	*P*
			(95%CI)	(95%CI)	(%)	(95%CI)	(95%CI)	(95%CI)	(95%CI)	
**ASC-US**	OCT	CIN2+	70.4(56.2,81.6)	77.2(70.3,83.0)	75.6	48.1(36.8,59.6)	89.7(83.5,93.8)	3.089(2.244,4.254)	0.384(0.254,0.581)	< 0.001^a^
		CIN3+	76.5(49.8,92.2)	69.6(62.9,75.5)	70.1	16.5(9.4,26.9)	97.4(93.1,99.2)	2.514(1.804,3.503)	0.338(0.143,0.800)	< 0.001^a^
	hrHPV	CIN2+	98.1(88.8,99.9)	18.3(13.1,24.9)	36.8	26.5(20.6,33.3)	97.1(82.9,99.8)	1.202(1.111,1.300)	0.101(0.014,0.739)	< 0.001^b^
		CIN3+	100(77.1,100)	15.7(11.2,21.4)	21.8	8.5(5.2,13.5)	100(87.4,100)	1.186(1.120,1.256)	\	< 0.001^b^
	OCT and hrHPV^†^	CIN2+	68.5(54.3,80.1)	82.2(75.7,87.4)	79.1	53.6(41.3,65.6)	89.7(83.8,93.7)	3.854(2.682,5.538)	0.383(0.258,0.569)	0.002^c^
		CIN3+	76.5(49.8,92.2)	74.2(67.7,79.8)	74.4	18.8(10.8,30.4)	97.6(93.5,99.2)	2.963(2.094,4.193)	0.317(0.134,0.750)	0.334^c^
	OCT or hrHPV^‡^	CIN2+	100(91.7,100)	13.3(8.9,19.4)	33.3	25.7(20.1,32.3)	100(82.8,100)	1.154(1.090,1.222)	\	0.002^d^
		CIN3+	100(77.1,100)	11.1(7.4,16.2)	17.5	8.1(4.9,12.9)	100(82.8,100)	1.124(1.073,1.178)	\	0.334^d^
**LSIL**	OCT	CIN2+	72.7(54.2,86.1)	78.0(67.3,86.1)	76.5	57.1(41.1,71.9)	87.7(77.4,93.9)	3.313(2.095,5.240)	0.349(0.199,0.614)	< 0.001 ^a^
		CIN3+	73.3(44.8,91.1)	69.0(58.9,77.7)	69.6	26.2(14.4,42.3)	94.5(85.8,98.2)	2.366(1.550,3.610)	0.386(0.166,0.902)	< 0.001 ^a^
	hrHPV	CIN2+	90.9(74.5,97.6)	9.8(4.6,18.8)	33.0	28.8(20.6,38.7)	72.7(39.3,92.7)	1.007(0.885,1.146)	0.932(0.254,3.419)	< 0.001 ^b^
		CIN3+	86.7(58.4,97.7)	9.0(4.5,16.8)	19.1	12.5(7.1,20.8)	81.8(47.8,96.8)	0.952(0.774,1.172)	1.481(0.334,6.563)	< 0.001 ^b^
	OCT and hrHPV^†^	CIN2+	66.7(48.1,81.4)	78.0(67.3,86.1)	74.8	55(38.7,70.4)	85.3(74.8,92.1)	3.037(1.890,4.879)	0.427(0.262,0.697)	0.500 ^c^
		CIN3+	60(32.9,82.5)	69(58.9,77.7)	67.8	22.5(11.4,38.9)	92(82.8,96.7)	1.935(1.167,3.211)	0.580(0.309,1.087)	0.782 ^c^
	OCT or hrHPV^‡^	CIN2+	97.0(82.5,99.8)	9.8(4.6,18.8)	34.8	30.2(21.9,40.0)	88.9(50.7,99.4)	1.075(0.979,1.180)	0.311(0.036,2.670)	0.500 ^d^
		CIN3+	100(74.7,100)	9(4.5,16.8)	20.9	14.2(8.4,22.6)	100(62.9,100)	1.099(1.033,1.169)	\	0.782 ^d^
**Minor abnormal cervical cytology**	OCT	CIN2+	71.3(60.4,80.2)	77.5(71.8,82.3)	75.9	51.2(42.0,60.4)	89.0(84.1,92.6)	3.165(2.437,4.109)	0.371(0.266,0.517)	< 0.001 ^a^
		CIN3+	75(56.2,87.9)	69.4(64.0,74.4)	69.9	19.8(13.4,28.3)	96.5(92.9,98.4)	2.451(1.890,3.178)	0.360(0.197,0.658)	< 0.001 ^a^
	hrHPV	CIN2+	95.4(88.0,98.5)	15.6(11.6,20.8)	35.5	27.3(22.4,32.7)	91.1(77.9,97.1)	1.131(1.055,1.213)	0.294(0.108,0.797)	< 0.001 ^b^
		CIN3+	93.8(77.8,98.9)	13.6(10.1,17.9)	20.9	9.9(6.9,13.9)	95.6(83.6,99.2)	1.085(0.982,1.198)	0.461(0.116,1.834)	< 0.001 ^b^
	OCT and hrHPV^†^	CIN2+	67.8(56.8,77.2)	80.9(75.5,85.4)	77.7	54.1(44.3,63.6)	88.3(83.4,92.0)	3.554(2.664,4.741)	0.398(0.293,0.541)	< 0.001 ^c^
		CIN3+	68.8(49.9,83.3)	72.6(67.2,77.3)	72.2	20.2(13.3,29.2)	95.8(92.2,97.9)	2.505(1.866,3.362)	0.431(0.257,0.722)	0.341 ^c^
	OCT or hrHPV^‡^	CIN2+	98.9(92.9,100)	12.2(8.6,16.9)	33.8	27.2(22.5,32.5)	97.0(82.5,99.8)	1.126(1.071,1.184)	0.094(0.013,0.697)	< 0.001 ^d^
		CIN3+	100(86.7,100)	10.4(7.4,14.4)	18.6	10.1(7.1,14.1)	100(87.0,100)	1.116(1.075,1.159)	\	0.341 ^d^

OCT, optical coherence tomography; hrHPV, high-risk human papillomavirus; ASC-US, atypical squamous cells of undetermined significance; LSIL, low-grade squamous intraepithelial lesion; PPV, positive predictive value; NPV, negative predictive value; +LR, positive likelihood ratio; -LR, negative likelihood ratio; 95%CI, 95% confidence interval.

^†^, “and”: the AND rule, in which the diagnosis is positive only if both A and B are positive. Either A or B can be negative for the diagnosis to be negative, trust negative.

^‡^, “or”: the OR rule, in which the diagnosis is positive if either A or B is positive. Both A and B must be negative for the diagnosis to be negative, trust positive.

^a^, OCT vs. hrHPV testing.

^b^, OCT and hrHPV testing vs. hrHPV testing.

^c^, OCT vs. OCT and hrHPV testing.

^d^, OCT or hrHPV testing vs. hrHPV testing.

### The performance of OCT triage in minor abnormal cervical cytology

According to the 2019 American Society of Colposcopy and Cervical Pathology (ASCCP) management consensus guideline, colposcopy is recommended for women with cervical cancer screening results of hrHPV-positive ASC-US and LSIL.

To further investigate the efficiency of OCT triage in such women, we studied the sensitivity, specificity, accuracy, PPV, NPV, +LR and -LR of OCT for detecting CIN2+/CIN3+ combined with hrHPV testing (Table 4 and S1 Table).

**Table 4:** The performance of OCT for detecting CIN2+/CIN3+ combined with hrHPV testing.

TCT result	hrHPV test result		sensitivity(%)	specificity(%)	Accuracy	PPV(%)	NPV(%)	+LR(%)	-LR(%)
			(95%CI)	(95%CI)	(%)	(95%CI)	(95%CI)	(95%CI)	(95%CI)
**ASC-US**	hrHPV (+)	CIN2+	69.8(55.5,81.3)	78.2(70.5,84.4)	76	53.6(41.3,65.6)	87.8(80.6,92.6)	3.207(2,251,4.569)	0.386(0.255,0.583)
		CIN3+	76.5(49.8,92.2)	69.4(62.1,75.9)	70	18.8(10.8,30.4)	96.9(91.9,99.0)	2.499(1.775,3.519)	0.339(0.143,0.803)
	HPV16/18 (+)	CIN2+	81.8(59.0,94.0)	91.7(71.5,98.5)	87.0	90(66.9,98.2)	84.6(64.3,95.0)	9.818(2.567,37.550)	0.198(0.081,0.485)
		CIN3+	88.9(50.7,99.4)	67.6(50.1,81.4)	71.7	40(20.0,63.6)	96.2(78.4,99.8)	2.741(1.631,4.607)	0.164(0.025,1.070)
	HPV16/18 (-)^§^	CIN2+	61.3(42.3,77.6)	75.6(66.9,82.7)	72.7	38.8(25.5,53.8)	88.6(80.5,93.7)	2.513(1.654,3.819)	0.512(0.327,0.801)
		CIN3+	62.5(25.9,89.8)	69.9(61.6,77.0)	69.5	10.2(3.8,23.0)	97.1(91.3,99.3)	2.074(1.149,3.744)	0.537(0.218,1.321)
	hrHPV (-)	CIN2+	100(5.5,100)	72.7(54.2,86.1)	73.5	10(0.5,45.9)	100(82.8,100)	3.667(2.100,6.401)	\
		CIN3+	\	70.6(52.3,84.3)	70.6	0(0,34.5)	100(82.8,100)	\	\
**LSIL**	hrHPV (+)	CIN2+	73.3(53.8,87.0)	75.7(64.1,84.6)	75	55(38.7,70.4)	87.5(76.3,94.1)	3.015(1.911,4.757)	0.352(0.193,0.643)
		CIN3+	69.2(38.9,89.6)	65.9(55.2,75.3)	66.3	22.5(11.4,38.9)	93.8(84.0,98.0)	2.032(1.281,3.224)	0.467(0.204,1.066)
	HPV16/18 (+)	CIN2+	75(35.6,95.5)	78.6(48.8,94.3)	77.3	66.7(30.9,91.0)	84.6(53.7,97.3)	3.5(1.189,10.305)	0.318(0.092,1.098)
		CIN3+	80(29.9,98.9)	70.6(44.0,88.6)	72.7	44.4(15.3,77.3)	92.3(62.1,99.6)	2.72(1.154,6.408)	0.283(0.047,1.716)
	HPV16/18 (-)^§^	CIN2+	72.7(49.6,88.4)	75(61.9,84.9)	74.4	51.6(33.4,69.4)	88.2(75.4,95.1)	2.909(1.751,4.832)	0.364(0.182,0.727)
		CIN3+	37.5(10.2,74.1)	35.1(24.6,47.2)	64.6	5.9(1.5,17.2)	83.9(65.5,93.9)	0.578(0.233,1.437)	1.779(0.990,3.195)
	hrHPV (-)	CIN2+	66.7(12.5,98.2)	100(59.8,100)	90.9	100(19.8,100)	88.9(50.7,99.4)	\	0.333(0.067,1.652)
		CIN3+	100(19.8,100)	100(62.9,100)	100	100(19.8,100)	100(62.9,100)	\	\

OCT: optical coherence tomography. hrHPV: high-risk human papillomavirus. HPV16/18, HPV-16 or HPV-18; ASC-US, atypical squamous cells of undetermined significance; LSIL, low-grade squamous intraepithelial lesion; PPV, positive predictive value; NPV, negative predictive value; +LR, positive likelihood ratio; -LR, negative likelihood ratio; 95%CI, 95% confidence interval.

^§^, HPV16/18 (-): hrHPV positive result excluded HPV-16 and HPV-18 positive result.

When ASC-US cytology with positive hrHPV, the NPV of OCT for detecting CIN2+/CIN3+ was 87.8%/96.9%, -LR was 0.386/0.339, and the sensitivity, specificity, accuracy, PPV and +LR were 69.8%/76.5%, 78.2%/69.4%, 76%/70%, 53.6%/18.8% and 3.207/2.499, respectively. We further analyzed the data according to HPV genotype. Only NPV and -LR for CIN2+/CIN3+ detection was low in positive HPV 16/18 cases compared with positive non-HPV16/18 cases (84.6%/96.2% vs. 88.6%/97.1%; 0.198/0.164 vs. 0.512/0.537). When ASC-US cytology with negative hrHPV, NPVs of OCT for detecting CIN2+ and CIN3+ were both 100%.

When LSIL cytology with negative hrHPV, the sensitivity, specificity, accuracy, PPV, NPV and -LR of OCT for detecting CIN2+ were 66.7%, 100%, 90.9%,100%, 88.9% and 0.333, respectively. We demonstrated valuable results in identifying CIN3+ cases. The sensitivity, specificity, accuracy, PPV and NPV of OCT were all 100%.

### The value of OCT in combined with cervical screening

The colposcopy referral rates of OCT were lower than those of hrHPV testing in TCT results of ASC-US and LSIL (33.8% and 36.5% of OCT, 85.5% and 90.4% of hrHPV testing, *P* <0.001) (Table 5). Therefore, the colposcopy referral rate based on OCT classification was lower than that based on hrHPV testing in minor abnormal TCT(34.7% vs. 87.1%, *P* <0.001).

**Table 5:** Colposcopy referral rate of different screening methods.

TCT result	Screening method	hrHPV test result	Colposcopy referral rate (%)	*P*
**ASC-US**	hrHPV		85.5 (200/234)	< 0.001
	OCT		33.8 (79/234)	
		hrHPV (+)	34.5 (69/200)	
		HPV16/18 (+)	43.5 (20/46)	
		HPV16/18 (-)^§^	31.8 (49/154)	
		hrHPV (-)	29.4 (10/34)	
**LSIL**	hrHPV		90.4 (104/115)	< 0.001
	OCT		36.5 (42/115)	
		hrHPV (+)	38.5 (40/104)	
		HPV16/18 (+)	40.9 (9/22)	
		HPV16/18 (-)^§^	37.8 (31/82)	
		hrHPV (-)	18.2 (2/11)	
**Minor abnormal cervical cytology**	hrHPV		87.1 (304/349)	< 0.001
	OCT		34.7 (121/349)	
		hrHPV (+)	35.9 (109/304)	
		HPV16/18 (+)	42.6 (29/68)	
		HPV16/18 (-)^§^	33.9 (80/236)	
		hrHPV (-)	26.7 (12/45)	

OCT, optical coherence tomography; hrHPV, high-risk human papillomavirus; HPV16/18, HPV-16 or HPV-18; ASC-US, atypical squamous cells of undetermined significance; LSIL, low-grade squamous intraepithelial lesion.

^^^, HPV16/18 (-): hrHPV positive result excluded HPV-16 and HPV-18 positive result.

**Fig 3:**
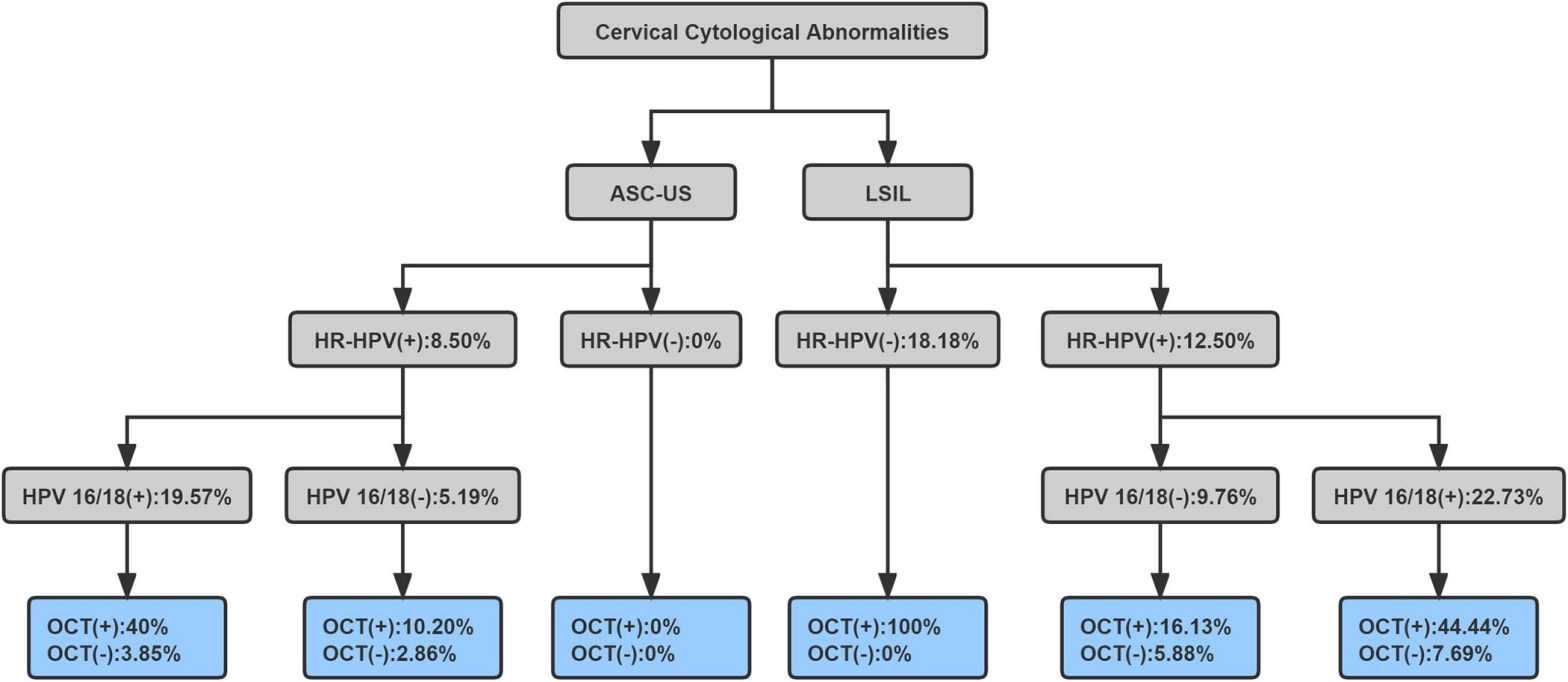
The immediate risk of CIN3+ of different screening methods. ASC-US, atypical squamous cells of undetermined significance; LSIL, low-grade squamous intraepithelial lesion; hrHPV, high-risk human papillomavirus; HPV16/18, HPV-16 or HPV-18 positive result; OCT, optical coherence tomography.

In order to better manage the women with minor abnormal cervical cytology, we calculated the immediate risk of CIN2+/CIN3+ of different screening methods (Fig 3, S2 Table and Table 6). The immediate risk of CIN2+ was zero in hrHPV-negative ASC-US cases with OCT negative results. According to the 2019 ASCCP guidelines, patients with an immediate CIN3+ risk of 4% or more should undergo a colposcopy, and patients with an immediate CIN3+ risk of 0.55%-4% will be reviewed after one year. The study found patients with hrHPV-positive ASC-US, the immediate CIN3+ risk was less than 4% in OCT negative cases (3.85% or 2.86%). The immediate risk of CIN3+ was zero in hrHPV-negative LSIL cases with OCT negative results, however, the risk was more than 4% in cases with OCT positive results.

**Table 6:** The immediate risk of CIN3+ of different screening methods.

Different screening methods				Number (n)	CIN3+ (n)	Risk (%)	95%CI
**ASC-US**	hrHPV (+)			200	17	8.5	4.63–12.37
		hrHPV16/18 (+)		46	9	19.57	8.10–31.03
			OCT (+)	20	8	40	18.53–61.47
			OCT (-)	26	1	3.85	0–11.24
		hrHPV16/18 (-)^§^		154	8	5.19	1.69–8.70
			OCT (+)	49	5	10.20	1.73–18.68
			OCT (-)	105	3	2.86	0–6.04
	hrHPV (-)			34	0	0	0–0
			OCT (+)	10	0	0	0–0
			OCT (-)	24	0	0	0–0
**LSIL**	hrHPV (+)			104	13	12.50	6.14–18.86
		hrHPV16/18 (+)		22	5	22.73	5.22–40.24
			OCT (+)	9	4	44.44	11.98–76.91
			OCT (-)	13	1	7.69	0–22.18
		hrHPV16/18 (-)^§^		82	8	9.76	3.33–16.18
			OCT (+)	31	5	16.13	3.18–29.08
			OCT (-)	51	3	5.88	0–12.34
	hrHPV (-)			11	2	18.18	0–40.97
			OCT (+)	2	2	100	100–100
			OCT (-)	9	0	0	0–0

OCT, optical coherence tomography; hrHPV, high-risk human papillomavirus; HPV16/18, HPV-16 or HPV-18; ASC-US, atypical squamous cells of undetermined significance; LSIL, low-grade squamous intraepithelial lesion.

^§^, HPV16/18 (-): hrHPV positive result excluded HPV-16 and HPV-18 positive result.

**Fig 4:**
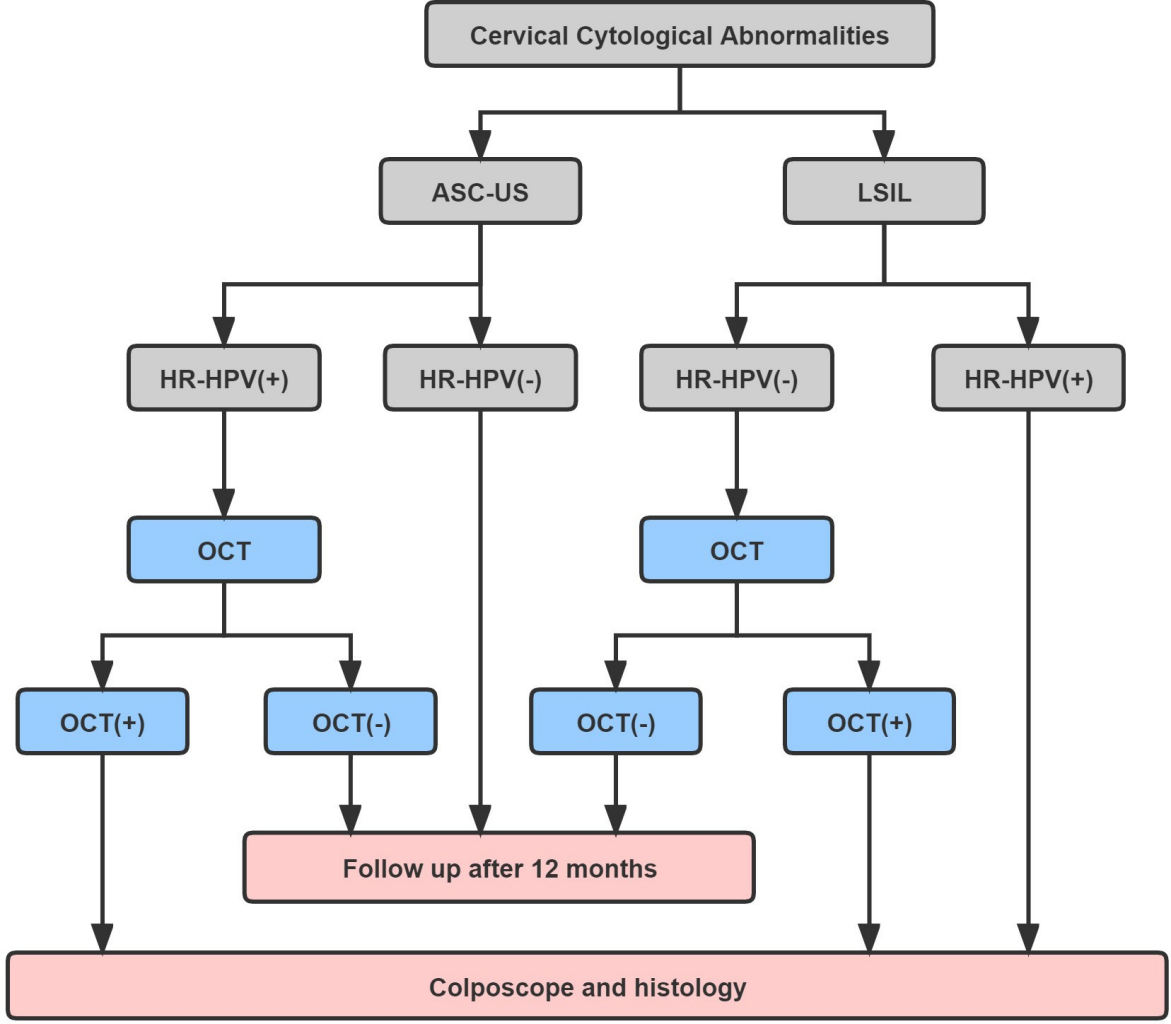
Recommendation of a novel method of OCT combined with cervical screening. ASC-US, atypical squamous cells of undetermined significance; LSIL, low-grade squamous intraepithelial lesion; hrHPV, high-risk human papillomavirus; HPV16/18, HPV-16 or HPV-18 positive result; OCT, optical coherence tomography.

These results indicated that women with minor abnormal cervical cytology can be better managed by OCT combined cervical screening before colposcopy. Therefore, we proposed a novel method of OCT combined with cervical screening and tried to reduce the colposcopy referral rate (Fig 4). Women with hrHPV positive ASC-US or hrHPV negative LSIL results can be determined whether the next step should be colposcopy or close follow-up by OCT examination.

## Discussion

Cervical cancer is a serious threat to women’s health in China. In 2020, an estimated 109,700 women were diagnosed with cervical cancer and about 59,000 women died from the disease in China. Therefore, the disease burden of cervical cancer is huge in China. The overall prevalence of hrHPV infection was 19.4% among 137,943 gynecological outpatients [19]. The high rate of hrHPV infection has brought great pressure on colposcopy examination in the second step protocols for cervical cancer screening. Our study provides a new non-invasive technique for reducing unnecessary colposcopy in co-testing of hrHPV and cytology. The OCT images are favorable for triage of minor abnormal cervical cytology ASC-US/LSIL.

Our study demonstrated that the high-resolution OCT images can identify the microstructures of normal and diseased cervix. OCT is often denoted as the optical analog of ultrasound and its resolution is 50–100 times finer than ultrasound [20]. Therefore, OCT images can easily show characteristic changes of benign lesions, such as the Nabothian cysts hidden in the subepithelial matrix, cervical epithelium of post-LEEP and atrophic vaginitis. The normal cervical OCT images exhibited a clear three-layered structure of cervical epithelium, basement membrane and stroma in the study. With the progress of cervical lesions, three-layered structure of the cervix becomes less distinct and regular. Quantitative analysis of OCT images can be used to noninvasively assess normal and abnormal cervical tissue in vivo [21]. The OCT images of suspected CIN patients had confirmed the potential of OCT in improving the differentiation of cervical cancer and precancerous lesions [11]. These studies supported our results.

We evaluated the diagnostic efficiency of OCT in women with abnormal cervical cytology. According to the characteristics of OCT images, our study took CIN2+ as the positive standard of OCT. The specificity, accuracy and PPV of OCT for detecting CIN2+/CIN3+ were all higher than those of hrHPV testing (77.5%/69.4% vs. 15.6%/13.6%, 75.9%/69.9% vs. 35.5%/20.9%, 51.2%/19.8% vs. 27.3%/9.9%, respectively). Other studies also had evaluated the diagnostic efficacy of OCT in cervical lesions. When the positive threshold of histopathology was set to CIN1, the sensitivity and specificity were 96%-98% and 39%-41% respectively; when the threshold was set to CIN2, the sensitivity and specificity were 84%-86% and 60%-64% respectively [12]. The research of Ren found that OCT had a good sensitivity of 87.0% (95%CI: 82.2–90.7) and a specificity of 84.1% (95%CI: 80.3–87.2) for detecting cervical lesions [14]. The method of OCT image analysis in our study was consistent with Ren. All the above studies confirmed OCT is valuable for the in-vivo evaluation of cervical lesions. The characteristic of our study was to compare the diagnostic value of OCT with hrHPV testing in women with ASC-US/LSIL cytology. The results demonstrated the method of OCT positive result or positive hrHPV testing had highest sensitivity and NPV among different screening methods to detect CIN2+/CIN3+.

Current recommended primary cervical screening strategies are cytology, hrHPV testing, or co-testing [3]. The randomized trial of 10,154 participants showed that HPV test (HC2) was more sensitive to detect CIN2 than conventional cytology (Pap) (94.6% vs. 55.4%) [22]. The lower specificity and PPV of hrHPV testing may lead to an increase in colposcopy referrals and overtreatment. Compared with liquid-based cytology (LBC) alone, LBC with hrHPV testing triage had similar sensitivity, but had significantly increased specificity for CIN2+/CIN3+ [6]. Therefore, from the perspective of diagnostic efficiency, cytology combined with hrHPV testing is the best choice for cervical cancer screening. However, there is still controversial in the management of ASC-US and LSIL [7].

According to the 2019 ASCCP management consensus guideline, colposcopy is recommended for women with hrHPV-positive ASC-US cytology/LSIL cytology. The higher NPV and -LR of OCT in the study showed the value of detecting CIN2+/CIN3+ in cases with hrHPV-positive ASC-US cytology. The sensitivity, specificity, accuracy, PPV and NPV of OCT for identifying CIN3+ were all 100% in patients with hrHPV-negative LSIL cytology. The results confirmed the good performance of OCT was in these populations.

Our study found the colposcopy referral rate based on OCT classification was lower than that based on hrHPV testing in minor abnormal TCT results (34.7% vs. 87.1%, *P* <0.001). Further analysis showed that the colposcopy referral rates of OCT were 34.5% and 31.8% in women with hrHPV-positive ASC-US result and non-HPV16/18 positive ASC-US result. The colposcopy referral rates of OCT were 38.5%, 37.8% and 18.2% in women with hrHPV-positive LSIL result, non-HPV16/18 positive LSIL result and hr-HPV-negative LSIL result, respectively. These confirmed that OCT can reduce the colposcopy referral rate of women with minor abnormal cervical cytology result.

Our study has clinical significance in reducing the number of colposcopy in the second step of cervical cancer screening. Existing data showed that when women were diagnosed with ASC-US cytology or LSIL cytology by cervical screening, only 15.6% and 1.1% of cases or 11.9% and 0.3% of cases had final pathological diagnosis of ≥ CIN2+ and cervical cancer, respectively [8, 9]. Our study found that 23.1% and 28.7% of women with cervical cytology of ASC-US and LSIL had pathological diagnosis of ≥ CIN2+. Therefore, adding OCT to the joint screening of cytology and hrHPV testing may be valuable in reducing the workload of colposcopy. Especially in patients with hrHPV-positive ASC-US and hrHPV-negative LSIL result, the OCT negative patients can be followed up for one year according to the immediate risk of CIN3+.

One factor restricting the clinical promotion of OCT is that its image recognition needs special training. This requires a period of practice and experience. A researcher developed an automated algorithm to extract OCT image features and identity CIN2+. The algorithm achieved a sensitivity of 51% (95%CI: 36–67) and a specificity of 92% (95%CI: 86–96) [23]. This study brings better feasibility for the clinical application of OCT.

In our study, some results of OCT were inconsistent with corresponding pathological results. The reasons may be as follows: the location of the biopsy may be different from that of the OCT probe; the lesions were too small to be detected by OCT; more cervical mucus may affect the quality of OCT images and the accuracy of OCT image results. We found that the effect of mucus on OCT was more common during ovulation.

We found three disadvantages of OCT images at this stage. One was similar icicle-like features of OCT images of some squamous cell carcinoma (SCC) and HSIL. This made it difficult to distinguish OCT images of SCC and HSIL, but did not affect the classification of OCT. The second was the limitations of OCT examination. Two cases of cervical adenocarcinoma found by ECC were not detected by OCT alone. The two patients were characterized by type III cervical transformation area. The OCT probe cannot penetrate the cervical canal, and OCT cannot detect the lesions in the cervical canal. The third was the difficulty of OCT image recognition. OCT image recognition requires image classification experience and a period of image recognition training [13, 24].

In addition, our study had two limitations. One was participants all had abnormal TCT results. This may not represent the actual situation of the general population. In the future, the sample size and sample population need to be expanded to increase persuasiveness. OCT could be an adjunctive tool for minor abnormal cervical cytology, but its effectiveness still need to be improved. Artificial intelligence technology and large databases are being developed to improve OCT image recognizing [23]. The second was the design of the study. We provided hrHPV/TCT results to the OCT investigators. This may cause some subjective bias.

## Conclusions

OCT alone or in combination with hrHPV testing shows good performance for detecting CIN2+/CIN3+ in patients with ASC-US/LSIL cytology. OCT is an effective method for colposcopy triage in women with hrHPV positive ASC-US and hrHPV negative LSIL cytology.

## Supporting information

S1 Fig Low-risk OCT images and corresponding colposcopy images. (A) A case of chronic cervicitis with positive HPV58 test and ASC-US cytology, and pathological result was chronic cervicitis. OCT image showed squamous epithelial cells were arranged in a well-organized way forming a mesh-like structure, and a clear basement membrane was between the squamous epithelium and stroma. (B) A case of NC (chronic cervicitis) with positive HPV59 test and ASC-US cytology. OCT image showed epithelium layer became thinner due to the compression of the cyst, and dark areas of cystic liquid with clear boundaries in the stroma. (C) A case of cervical columnar epithelial ectropion (chronic cervicitis with scaling) had a positive HPV18, 53 and 66 with ASC-US cytology result. OCT image could not show normal squamous epithelial structure. The columnar epithelium cells formed regular papillary or glandular structures in ectropion tissue. (D) A case of LSIL (CIN1) had a positive HPV53 test with ASC-US cytology result. OCT image showed normal epithelium layer and partially or completely visible basement membrane. The koilocytotic cells showed enlarged nucleus and high scattering. (A1-D1) OCT images. (A2-D2) colposcopy images. EP, epithelium; ST, stroma; BM, basement membrane; NC, Nabothian cysts. OCT scale bars 200 μm. Colposcopy magnification: ×7.5.
(TIF)

S2 Fig High-risk OCT images and corresponding colposcopy images. (A) A case of HSIL (CIN2) had a positive HPV16 test with ASC-US cytology result. OCT image showed the epithelial layer and basement membrane were invisible, the overall refractive index was uneven, the surface layer was bright and the brightness decreased rapidly with depth. Icicle-like alternating light and dark shadows (arrows) were usually observed in HSIL cases. (B) A case of cervical squamous cell carcinoma had a positive HPV31 and 58 test with ASC-H cytology result. OCT image showed heterogeneous regions of hypo-scattering or hyper-scattering nests or clusters (circles) of squamous cell tumors. (A1-B1) OCT images. (A2-B2) colposcopy images. OCT scale bars 200 μm. Colposcopy magnification: ×7.5.
(TIF)

S3 Fig Special OCT images and corresponding colposcopy images. (A) A case after LEEP had a positive HPV58 test with LSIL cytology result. Her pathological result was CIN2. OCT image showed that epithelial layer covered almost the entire field of vision, and basement membrane was invisible or indistinct due to the thickness of epithelial layer. (B, C) A case of atrophic vaginitis (chronic cervicitis) had a positive HPV53 test with LSIL cytology result. OCT images of atrophic vaginitis showed the cervical epithelial layer was thin before estrogen treatment, and thicken after 3 weeks treatment. The thickness of cervical epithelial layer was about 2 times than that before treatment by Image J. (A1-C1) OCT images. (A2-C2) colposcopy images. EP, epithelium; ST, stroma; BM: basement membrane. OCT scale bars 200 μm. Colposcopy magnification: ×7.5.
(TIF)

S1 Table The performance of OCT for detecting CIN2+/CIN3+ combined with hrHPV testing. OCT: optical coherence tomography. hrHPV: high-risk human papillomavirus. HPV16/18, HPV-16 or HPV-18; PPV, positive predictive value; NPV, negative predictive value; +LR, positive likelihood ratio; -LR, negative likelihood ratio; 95%CI, 95% confidence interval. ^§^, HPV16/18 (-): hrHPV positive result excluded HPV-16 and HPV-18 positive result.
(DOCX)

S2 Table The immediate risk of CIN2+ of different screening methods. OCT, optical coherence tomography; hrHPV, high-risk human papillomavirus; HPV16/18, HPV-16 or HPV-18; ASC-US, atypical squamous cells of undetermined significance; LSIL, low-grade squamous intraepithelial lesion. ^§^, HPV16/18 (-): hrHPV positive result excluded HPV-16 and HPV-18 positive result.
(DOCX)
