# Supplementary figures and images for: Efficacy of optical coherence tomography in the triage of women with minor abnormal cervical cytology before colposcopy
Source: PLoS One. 2023 Mar 13;18(3):e0282833. doi: 10.1371/journal.pone.0282833 (PMC10010519; doi:10.1371/journal.pone.0282833)

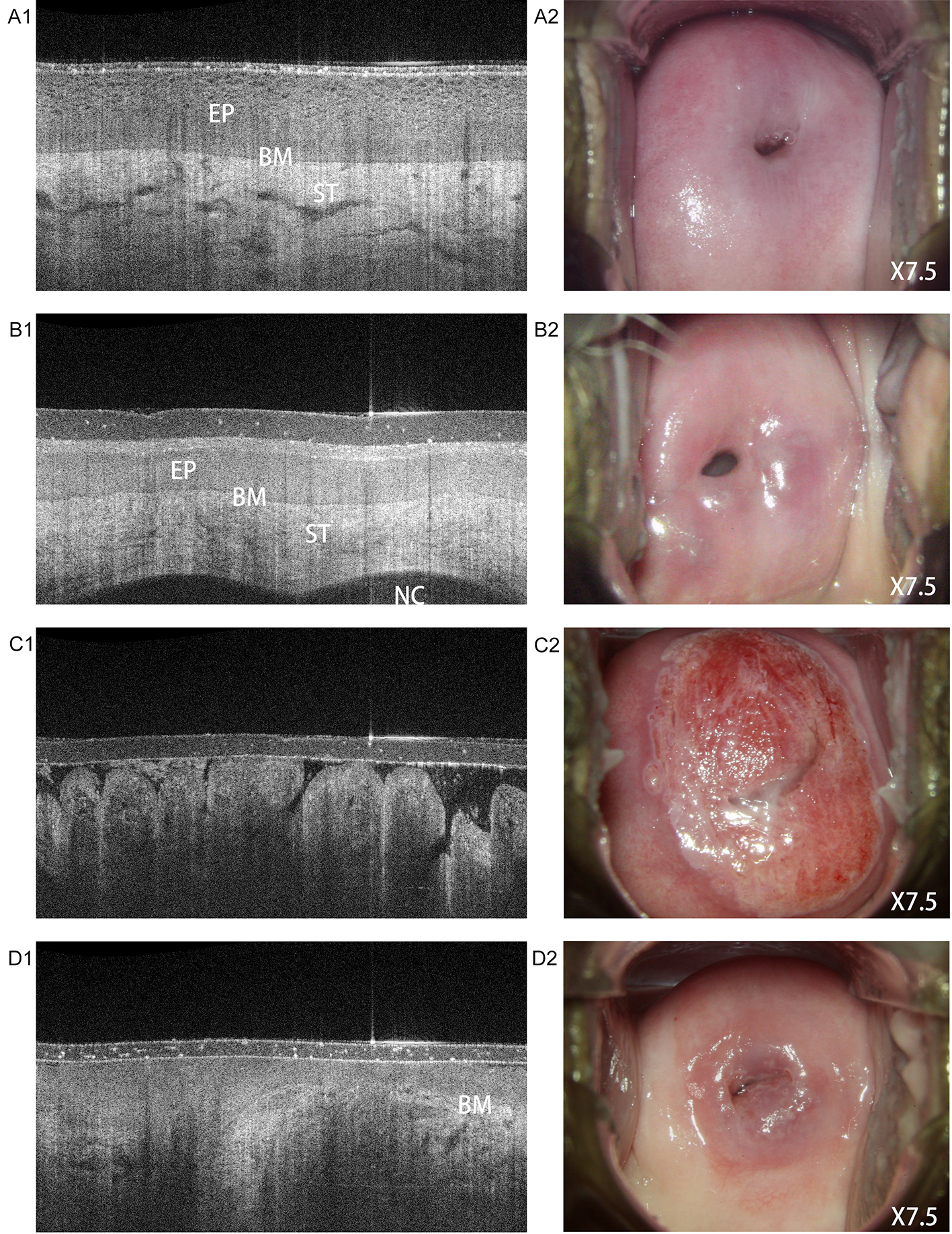

Supplement: S1 Fig — (A) A case of chronic cervicitis with positive HPV58 test and ASC-US cytology, and pathological result was chronic cervicitis. OCT image showed squamous epithelial cells were arranged in a well-organized way forming a mesh-like structure, and a clear basement membrane was between the squamous epithelium and stroma. (B) A case of NC (chronic cervicitis) with positive HPV59 test and ASC-US cytology. OCT image showed epithelium layer became thinner due to the compression of the cyst, and dark areas of cystic liquid with clear boundaries in the stroma. (C) A case of cervical columnar epithelial ectropion (chronic cervicitis with scaling) had a positive HPV18, 53 and 66 with ASC-US cytology result. OCT image could not show normal squamous epithelial structure. The columnar epithelium cells formed regular papillary or glandular structures in ectropion tissue. (D) A case of LSIL (CIN1) had a positive HPV53 test with ASC-US cytology result. OCT image showed normal epithelium layer and partially or completely visible basement membrane. The koilocytotic cells showed enlarged nucleus and high scattering. (A1-D1) OCT images. (A2-D2) colposcopy images. EP, epithelium; ST, stroma; BM, basement membrane; NC, Nabothian cysts. OCT scale bars 200 μm. Colposcopy magnification: ×7.5. (TIF) [file pone.0282833.s001.tif]

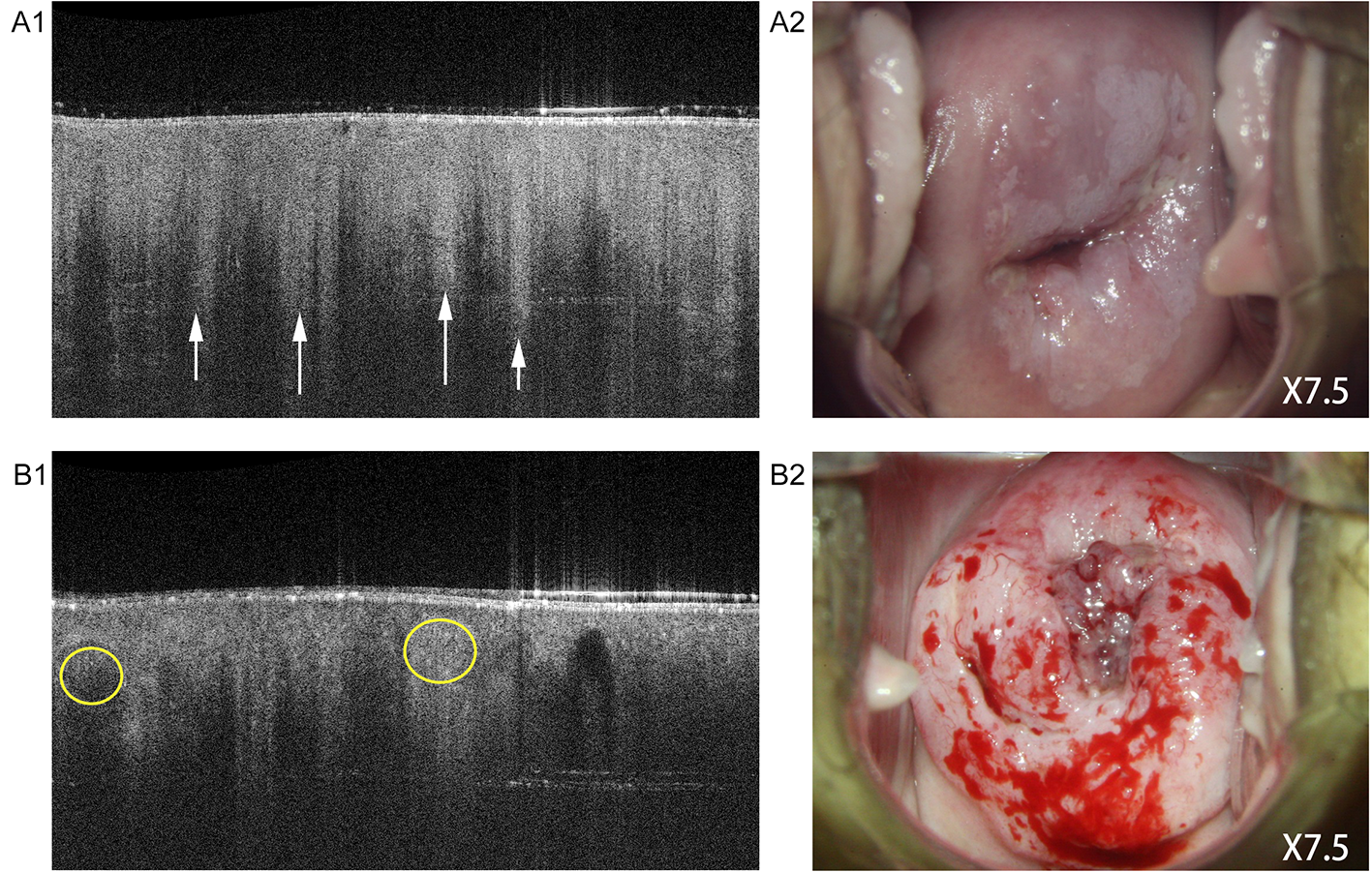

Supplement: S2 Fig — (A) A case of HSIL (CIN2) had a positive HPV16 test with ASC-US cytology result. OCT image showed the epithelial layer and basement membrane were invisible, the overall refractive index was uneven, the surface layer was bright and the brightness decreased rapidly with depth. Icicle-like alternating light and dark shadows (arrows) were usually observed in HSIL cases. (B) A case of cervical squamous cell carcinoma had a positive HPV31 and 58 test with ASC-H cytology result. OCT image showed heterogeneous regions of hypo-scattering or hyper-scattering nests or clusters (circles) of squamous cell tumors. (A1-B1) OCT images. (A2-B2) colposcopy images. OCT scale bars 200 μm. Colposcopy magnification: ×7.5. (TIF) [file pone.0282833.s002.tif]

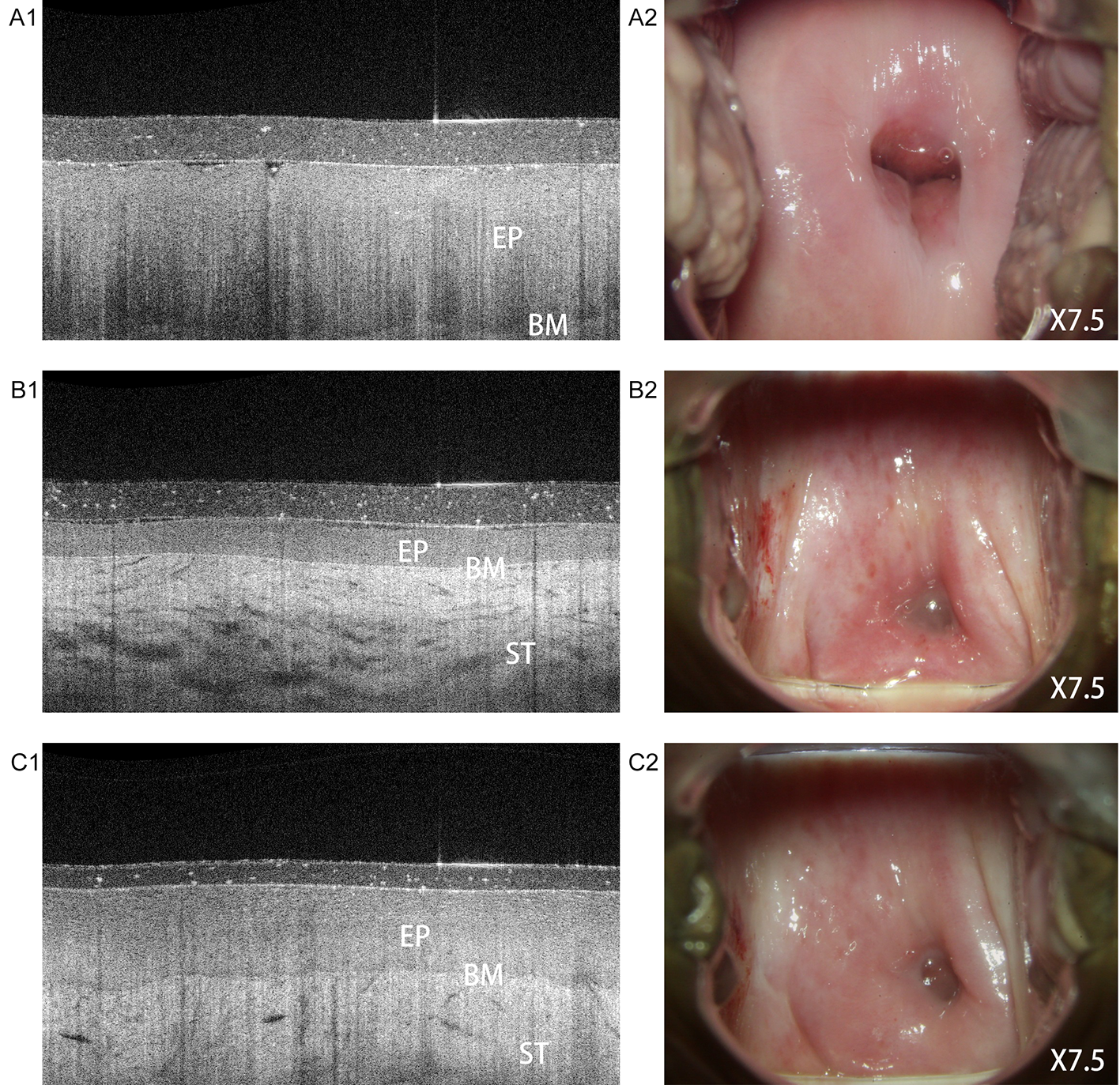

Supplement: S3 Fig — (A) A case after LEEP had a positive HPV58 test with LSIL cytology result. Her pathological result was CIN2. OCT image showed that epithelial layer covered almost the entire field of vision, and basement membrane was invisible or indistinct due to the thickness of epithelial layer. (B, C) A case of atrophic vaginitis (chronic cervicitis) had a positive HPV53 test with LSIL cytology result. OCT images of atrophic vaginitis showed the cervical epithelial layer was thin before estrogen treatment, and thicken after 3 weeks treatment. The thickness of cervical epithelial layer was about 2 times than that before treatment by Image J. (A1-C1) OCT images. (A2-C2) colposcopy images. EP, epithelium; ST, stroma; BM: basement membrane. OCT scale bars 200 μm. Colposcopy magnification: ×7.5. (TIF) [file pone.0282833.s003.tif]
