# Supplementary material for: Efficacy of optical coherence tomography in the triage of women with minor abnormal cervical cytology before colposcopy
Source: PLoS One. 2023 Mar 13;18(3):e0282833. doi: 10.1371/journal.pone.0282833 (PMC10010519; doi:10.1371/journal.pone.0282833)
Supplement: S1 Table — OCT: optical coherence tomography. hrHPV: high-risk human papillomavirus. HPV16/18, HPV-16 or HPV-18; PPV, positive predictive value; NPV, negative predictive value; +LR, positive likelihood ratio; -LR, negative likelihood ratio; 95%CI, 95% confidence interval. §, HPV16/18 (-): hrHPV positive result excluded HPV-16 and HPV-18 positive result. (DOCX) [file pone.0282833.s004.docx]

**S1 Table. The performance of OCT for detecting CIN2+/CIN3+ combined with hrHPV testing.**

| **TCT result** | **hrHPV test result** |  | **sensitivity(%)**  **(95%CI)** | **specificity(%)**  **(95%CI)** | **Accuracy**  **(%)** | **PPV(%)**  **(95%CI)** | **NPV(%)**  **(95%CI)** | **+LR(%)**  **(95%CI)** | **-LR(%)**  **(95%CI)** |
| --- | --- | --- | --- | --- | --- | --- | --- | --- | --- |
| **Minor abnormal cervical cytology** | hrHPV (+) | CIN2+ | 71.1(59.9,80.3) | 77.4(71.2,82.6) | 75.7 | 54.1(44.3,63.6) | 87.7(82.0,91.8) | 3.142(2.375,4.156) | 0.374(0.266,0.525) |
|  |  | CIN3+ | 73.3(53.8,87.0) | 68.2(62.3,73.6) | 68.8 | 20.2(13.3,29.2) | 95.9(91.8,98.1) | 2.310(1.751,3.047) | 0.391(0.215,0.710) |
|  | HPV16/18 (+) | CIN2+ | 80(60.9,91.6) | 86.8(71.1,95.1) | 83.8 | 83.8(63.5,93.5) | 84.6(68.8,93.6) | 6.08(2.635,14.030) | 0.230(0.112,0.475) |
|  |  | CIN3+ | 85.7(56.2,97.5) | 68.5(54.3,80.1) | 72.1 | 41.4(24.1,60.9) | 94.9(81.4,99.1) | 2.723(1.740,4.261) | 0.208(0.057,0.765) |
|  | HPV16/18 (-)^§^ | CIN2+ | 66.0(51.6,78.1) | 75.4(68.4,81.3) | 73.3 | 43.8(32.8,55.3) | 88.5(82.1,92.8) | 2.686(1.952,3.694) | 0.450(0.308,0.658) |
|  |  | CIN3+ | 62.5(35.9,83.7) | 68.2(61.5,74.2) | 67.8 | 12.5(6.5,22.2) | 96.2(91.4,98.4) | 1.964(1.283,3.008) | 0.55(0.291,1.040) |
|  | hrHPV (-) | CIN2+ | 75(21.9,98.7) | 78.0(62.0,88.9) | 77.8 | 25(6.7,57.2) | 97.0(82.5,99.8) | 3.417(1.523,7.667) | 0.320(0.058,1.772) |
|  |  | CIN3+ | 100(19.8,100) | 76.7(61.0,87.7) | 77.8 | 16.7(2.9,49.1) | 100(87.0,100) | 4.3(2.498,7.401) | \ |

OCT: optical coherence tomography. hrHPV: high-risk human papillomavirus. HPV16/18, HPV-16 or HPV-18; PPV, positive predictive value; NPV, negative predictive value; +LR, positive likelihood ratio; -LR, negative likelihood ratio; 95%CI, 95% confidence interval.

^§^, HPV16/18 (-): hrHPV positive result excluded HPV-16 and HPV-18 positive result.
