# Supplementary material for: Efficacy of optical coherence tomography in the triage of women with minor abnormal cervical cytology before colposcopy
Source: PLoS One. 2023 Mar 13;18(3):e0282833. doi: 10.1371/journal.pone.0282833 (PMC10010519; doi:10.1371/journal.pone.0282833)
Supplement: S2 Table — OCT, optical coherence tomography; hrHPV, high-risk human papillomavirus; HPV16/18, HPV-16 or HPV-18; ASC-US, atypical squamous cells of undetermined significance; LSIL, low-grade squamous intraepithelial lesion. §, HPV16/18 (-): hrHPV positive result excluded HPV-16 and HPV-18 positive result. (DOCX) [file pone.0282833.s005.docx]

**S2 Table. The immediate risk of CIN2+ of different screening methods.**

| **Different screening methods** | | | | **Number (n)** | **CIN2+ (n)** | **Risk (%)** | **95%CI** |
| --- | --- | --- | --- | --- | --- | --- | --- |
| **ASC-US** | hrHPV (+) |  |  | 200 | 53 | 26.5 | 20.38-32.62 |
|  |  | hrHPV16/18 (+) |  | 46 | 22 | 47.83 | 33.39-62.26 |
|  |  |  | OCT (+) | 20 | 18 | 90 | 76.85-100 |
|  |  |  | OCT (-) | 26 | 4 | 15.38 | 1.52-29.25 |
|  |  | hrHPV16/18 (-)^§^ |  | 154 | 31 | 20.13 | 13.80-26.46 |
|  |  |  | OCT (+) | 49 | 19 | 38.78 | 25.13-52.42 |
|  |  |  | OCT (-) | 105 | 12 | 11.43 | 5.34-17.51 |
|  | hrHPV (-) | |  | 34 | 1 | 2.94 | 0-8.62 |
|  |  |  | OCT (+) | 10 | 1 | 10 | 0-28.59 |
|  |  |  | OCT (-) | 24 | 0 | 0 | 0-0 |
| **LSIL** | hrHPV (+) |  |  | 104 | 30 | 28.85 | 20.14-37.55 |
|  |  | hrHPV16/18 (+) |  | 22 | 8 | 36.36 | 16.26-56.47 |
|  |  |  | OCT (+) | 9 | 6 | 66.67 | 35.87-97.47 |
|  |  |  | OCT (-) | 13 | 2 | 15.38 | 0-35.00 |
|  |  | hrHPV16/18 (-)^§^ |  | 82 | 22 | 26.83 | 17.24-36.42 |
|  |  |  | OCT (+) | 31 | 16 | 51.61 | 34.02-69.21 |
|  |  |  | OCT (-) | 51 | 6 | 11.76 | 2.92-20.61 |
|  | hrHPV (-) | |  | 11 | 3 | 27.27 | 0.95-53.59 |
|  |  |  | OCT (+) | 2 | 2 | 100 | 100-100 |
|  |  |  | OCT (-) | 9 | 1 | 11.11 | 0-31.64 |

OCT, optical coherence tomography; hrHPV, high-risk human papillomavirus; HPV16/18, HPV-16 or HPV-18; ASC-US, atypical squamous cells of undetermined significance; LSIL, low-grade squamous intraepithelial lesion.

^§^, HPV16/18 (-): hrHPV positive result excluded HPV-16 and HPV-18 positive result.
